# Amino acid secretion influences the size and composition of copper carbonate nanoparticles synthesized by ureolytic fungi

**DOI:** 10.1007/s00253-019-09961-2

**Published:** 2019-07-09

**Authors:** Feixue Liu, Laszlo Csetenyi, Geoffrey Michael Gadd

**Affiliations:** 1 Geomicrobiology Group, School of Life Sciences, University of Dundee, Dundee, DD1 5EH Scotland, UK; 2 Concrete Technology Group, Department of Civil Engineering, University of Dundee, Dundee, DD1 4HN Scotland, UK

**Keywords:** Fungi, Biomineralization, Copper carbonate, Nanoparticles, Amino acids

## Abstract

The ureolytic activity of *Neurospora crassa* results in an alkaline carbonate-rich culture medium which can precipitate soluble metals as insoluble carbonates. Such carbonates are smaller, often of nanoscale dimensions, than metal carbonates synthesized abiotically which infers that fungal excreted products can markedly affect particle size. In this work, it was found that amino acid excretion was a significant factor in affecting the particle size of copper carbonate. Eleven different amino acids were found to be secreted by *Neurospora crassa*, and l-glutamic acid, l-aspartic acid and l-cysteine were chosen to examine the impact of amino acids on the morphology and chemical composition of copper carbonate minerals. X-ray powder diffraction (XRPD), scanning electron microscopy (SEM), Fourier-transform infrared (FTIR) spectroscopy, thermogravimetric analysis (TGA) and X-ray photoelectron spectroscopy (XPS) were used to characterize the obtained copper carbonate samples. Copper carbonate nanoparticles with a diameter of 100–200 nm were produced with l-glutamic acid, and the presence of l-glutamic acid was found to stabilize these particles in the early phase of crystal growth and prevent them from aggregation. FTIR and TG analysis revealed that the amino acid moieties were intimately associated with the copper mineral particles. Component analysis of the final products of TG analysis of the copper minerals synthesized under various conditions showed the ultimate formation of Cu, Cu_2_O and Cu_2_S, suggesting a novel synthesis method for producing these useful Cu-containing materials.

**Electronic supplementary material:**

The online version of this article (10.1007/s00253-019-09961-2) contains supplementary material, which is available to authorized users.

## Introduction

Copper nanoparticles have been considered as a viable alternative to gold and silver nanoparticles due to lower cost, higher natural abundance and comparable electrical and thermal conductivity (Dabera et al. 2017; Kimber et al. 2018). Malachite (Cu_2_(OH)_2_CO_3_) is an important semi-precious mineral which has attracted extensive recent interest in various applications, for example, as coatings and catalysts (Gawande et al. 2016), and as an important precursor for the production of other Cu-bearing compounds such as CuO as well as Cu (Zhang et al. 2016). Malachite, in various morphologies, has been obtained by various synthetic routes, including spherical (RodrIguez-Clemente et al. 1994), hierarchical (where the structure is different at different length scales) (Xu and Xue 2005) and nanoscale (Saikia et al. 2013). The chemical and/or physical properties of such materials vary significantly as a function of crystal size, morphology and structure, even though these materials consist of the same chemical components (Hochella et al. 2008). The development of reliable and environmentally friendly technologies for the synthesis of malachite with controlled size and structure has therefore been attracting growing interest.

Compared with traditional chemical methods, biological synthesis can be effectively employed because of cheaper starting materials and simpler processes (Thakkar et al. 2010). Biological activities can influence metal speciation, toxicity and mobility, as well as mineral formation or dissolution (Gadd and Raven 2010). Many microorganisms, including bacteria, yeasts and filamentous fungi, are able to produce inorganic materials in the nanoscale intracellularly or extracellularly (Gadd 2010). Through redox transformations and metabolic activities where metabolites (oxalate, carbon dioxide etc.) can be excreted, fungi are very effective in the biomineralization of metals, i.e. conversion of free metal ions into a solid insoluble biomineral form (Gadd 2007; Li et al. 2015). For example, the filamentous fungus *Neurospora crassa* has been successfully used for the synthesis of silver and gold nanoparticles intracellularly (Castro-Longoria et al. 2011) and cadmium carbonate nanoparticles extracellularly (Li et al. 2014). In previous studies, *Neurospora crassa* was found to be able to produce copper carbonate nanoparticles through the reaction of copper ions and carbonate ions which were released from urea hydrolysis by the enzyme amidohydrolase urease (Li and Gadd 2017):

1 $$ {\mathrm{NH}}_2{\mathrm{CO}\mathrm{NH}}_2+{2\mathrm{H}}_2\mathrm{O}\to {{2\mathrm{NH}}_4}^{+}+{{\mathrm{CO}}_3}^{2-} $$

2 $$ {\mathrm{H}}_2\mathrm{O}+{\mathrm{Cu}}^{2+}+{{\mathrm{CO}}_3}^{2-}\to {\mathrm{Cu}\mathrm{CO}}_3{\left(\mathrm{OH}\right)}_{\mathrm{n}}\ \left(\mathrm{s}\right)\downarrow $$

The use of filamentous fungi for nanoparticle synthesis represents a promising approach since fungi are able to secrete large amounts of chemical substances, for example, organic acids, amino acids and enzymes that may be involved in nanomaterial synthesis. The impact of such biological organic material on the biomineralization of carbonate minerals has been observed with both natural environmental samples and in laboratory studies (Braissant et al. 2003). Minerals frequently show a different ultrastructure and physico-chemical features in the presence of an organic matrix which can affect key events in mineral formation like nucleation and crystal growth stages (Briegel and Seto 2012; Jack et al. 2007; Jiang et al. 2017; Ngwenya et al. 2014). For example, Jiang et al. (2017) found that vaterite nanoparticles having a counterclockwise spiralling morphology could be induced by l-enantiomers of aspartic acid and glutamic acid, whereas a clockwise morphology was induced by d-enantiomers. However, there are very few reports on the impact of amino acids excreted by microorganisms on biomineralization of copper carbonate minerals and their potential industrial applications. The filamentous fungal growth form may also provide a template or scaffold for biomineral production that can be used to investigate the physical, chemical and biological properties of novel nanoparticles.

In this study, the influence of three amino acids (l-glutamic acid, l-aspartic acid and l-cysteine), which can be secreted by fungi, on biomineral formation was investigated. The chemical composition, morphology and thermal stability of copper carbonate synthesized by biomineralization, chemical synthesis in the presence of various amino acids, and inorganically synthesized minerals were investigated in order to characterize the mechanisms involved.

## Materials and methods

### Organism and media

The experimental fungus used in this study was *Neurospora crassa* (FGSC: 2489, Fungal Genetics Stock Centre (FGSC), Kansas, USA). It was routinely maintained on malt extract agar (MEA, Lab M limited, Bury, Lancashire, UK) in 90-mm diameter Petri dishes and grown at 25 °C in the dark. A urea-modified AP1 medium was used as the liquid media consisting of 2% (*w*/*v*) d-glucose (Merck, Readington Township, NJ, USA), 40 mM urea (Sigma-Aldrich, St. Louis, MO, USA), 4 mM K_2_HPO_4_∙3H_2_O (Sigma-Aldrich, USA), 0.8 mM MgSO_4_∙7H_2_O (Sigma-Aldrich, USA), 0.2 mM CaCl_2_∙6H_2_O (Sigma-Aldrich, USA), 1.7 mM NaCl (Sigma-Aldrich, USA), 9 × 10^−3^ mM FeCl_3_∙6H_2_O (Sigma-Aldrich, USA) and trace metals 0.014 mM ZnSO_4_∙7H_2_O (VWR, Radnor, PA, USA), 0.018 mM MnSO_4_∙4H_2_O (Sigma-Aldrich, USA) and 1.6 × 10^−3^ mM CuSO_4_∙5H_2_O (VWR, USA) (Li et al. 2014). After 3 days growth in the full AP1 medium, fungal biomass was collected and washed twice in sterile MilliQ water after centrifugation (× 4000*g*, 30 min), and continued to be incubated in a sterile phosphate-free AP1 medium for 12 days. The initial pH of AP1 medium was adjusted to pH 5.5 using 1 M HCl after autoclaving. All experiments were conducted at least in triplicate.

### Determination of amino acid concentrations

One millimolar single amino acid (aspartic acid, glutamic acid, alanine, proline and cysteine, respectively) solutions and Dulbecco’s modified Eagle’s medium (DMEM) (Sigma-Aldrich, USA) were prepared separately as standards. Fifty microlitres of samples and standards was collected and analysed for the free amino acid concentration by high-performance liquid chromatography (HPLC) after derivatization. Protein was precipitated from each sample by adding 200 μl trifluoroacetic acid (TFA) and methanol solution (volume ratio = 1:10) and centrifuged at × 20,000*g* for 10 min. The free amino acids were then eluted and concentrated from sample supernatants by sodium acetate, methanol, triethanolamine (TEA) solution (volume ratio = 2∶2:1), methanol, MilliQ water, TEA, phenylisothiocyanate (PITC) solution (volume ratio = 7∶1:1∶1) and methanol. Samples were dried thoroughly between each step at 46 °C in a rotary evaporator, and resuspended in eluent buffer (95% solution of 10 mL/L TEA and 150 mM sodium acetate (pH = 6.4), and 5% acetonitrile) and separated using a Hewlett Packard 1050 HPLC system (Minneapolis, Minnesota, USA) with post-column UV detection (254 nm). Final results were analysed using Clarity Lite software, and all samples and standards were conducted at least in triplicate (Poncet et al. 2014).

### Preparation of copper carbonate minerals

In this study, copper carbonate minerals synthesized in the different reaction systems were compared in order to characterize the role of amino acids in biomineralization. Copper carbonate minerals were prepared (1) from biomass-free fungal growth supernatants, (2) by chemical synthesis without the addition of amino acids as a control and (3) copper precipitates obtained by chemical synthesis with the addition of glutamic acid, aspartic acid and cysteine separately at different concentrations.

#### Copper carbonate nanoparticles produced in fungal growth supernatants

After 12 days growth, *N. crassa* growth supernatant was collected by centrifugation (× 4000*g*, 30 min). CuCl_2_ solution at a 20mM final concentration was added dropwise to the growth supernatant of *N. crassa*, and samples were placed on a roller shaker (60 rpm) overnight. Precipitated products were collected and washed twice with MilliQ water after centrifugation (× 10,000*g*, 30 min).

#### Copper carbonate minerals produced by chemical synthesis

Chemical synthesized copper carbonate minerals were used as a control and obtained by mixing 20 mM ammonium carbonate and 20 mM copper chloride solutions. In order to identify the impact of amino acids on copper carbonate formation, amino acids that were detected in fungal growth supernatants (glutamic acid, aspartic acid and cysteine) were also added into the chemical reaction mixture as additives to final concentrations of 0.2, 1 and 10 mM. The samples were mixed overnight on a roller shaker (60 rpm), and after collection, the minerals precipitated were examined by SEM, XRPD, FTIR and TGA to investigate the influence of amino acids on their nucleation, morphology and growth.

### Characterization of copper carbonate minerals

#### Scanning electron microscopy and energy-dispersive X-ray analysis

Scanning electron microscopy (SEM) images were obtained by using a field emission scanning electron microscope (FESEM) (Jeol JSM7400F). Dry samples were sputter coated with 5 nm gold and platinum using a Cressington 208HR sputter coater (Ted Pella, Inc., Redding, CA, USA). The chemical compositions were analysed by an energy-dispersive X-ray spectrometer (EDXA) (Oxford Instruments, Inca, Abingdon, Oxfordshire, UK). The particle size distribution histograms of nanoparticles were calculated by measuring 150 random particles using Nano Measurer 1.2.5. software.

#### X-ray powder diffraction analysis

X-ray powder diffraction (XRPD) patterns were obtained with Cu-Kα radiation using a Panalytical X-pert Pro diffractometer. The measurements were made using a step-scanning program with 0.02° per step in the range from 0 to 100°. XRPD data were analysed by reference to patterns in the International Centre for Diffraction Data Powder Diffraction File (PDF) for identification of the crystalline phases.

#### Attenuated total reflectance Fourier-transform infrared spectroscopy

The attenuated total reflectance Fourier-transform infrared (ATR-FTIR) spectra of samples in the form of powders were obtained using a Bruker Vertex 70 FTIR spectrometer. All spectra were measured in the wavelength range from 400 to 4000 cm^−1^, with a 4 cm^−1^ spectral resolution.

#### X-ray photoelectron spectroscopy

X-ray photoelectron spectroscopy (XPS) was performed using a Scienta ESCA-300 instrument (Scienta AB, Uppsala, Sweden) fitted with a non-monochromatic Al-Kα X-ray source. The survey (wide) spectra was collected from 1200 to 0 eV with a step size of 0.2 eV, and more detailed scans for elements C, O, N and Cu were performed over the regions of interest. CasaXPS software was used to analyse the XPS spectra core-level lines for curve fitting. All spectra were referenced to the O 1s peak of carbonate at 530.9 eV.

#### Thermodynamic modelling by Geochemist’s Workbench (GWB)

Solubility diagrams for biomineralization and copper-complex production in the presence of cysteine were obtained using GWB (https://www.gwb.com/index.php). The main ionic species and their activities were generated from software SpecE8, based on initial concentrations of the components in AP1 medium and solutions for copper-cysteine reaction. The software Act2 was used to construct solubility diagrams, and both SpecE8 and Act2 are included in the GWB software package. The Visual Minteq’s thermodynamic database was used for this study, but modified to include the reactions of copper and cysteine (L) (Berthon 1995):

3 $$ {\mathrm{Cu}\mathrm{L}}_2\leftrightharpoons {\mathrm{Cu}}^{2+}+{2\mathrm{L}}^{-}\kern2em \mathrm{L}\mathrm{og}\ \mathrm{K}=-16 $$

4 $$ \mathrm{CuL}\leftrightharpoons {\mathrm{Cu}}^{+}+{\mathrm{L}}^{-}\kern3.2em \mathrm{Log}\ \mathrm{K}=-19.19 $$

Log K is referred to stability constant of the complexes. All thermodynamic modelling was conducted at ambient temperature (25 °C).

#### Thermal properties

Thermogravimetric analysis (TGA) was used to assess the thermal stabilities of the copper minerals produced in the presence of amino acids using a Shimadzu TGA 50. About 50 mg mineral samples were heated from 20 to 1000 °C at a uniform nitrogen flow rate of 100 ml min^−1^. The chemical components of the final products after thermal decomposition were analysed by XRPD.

## Results

### Characterization of copper carbonate minerals

**Fig. 1:**
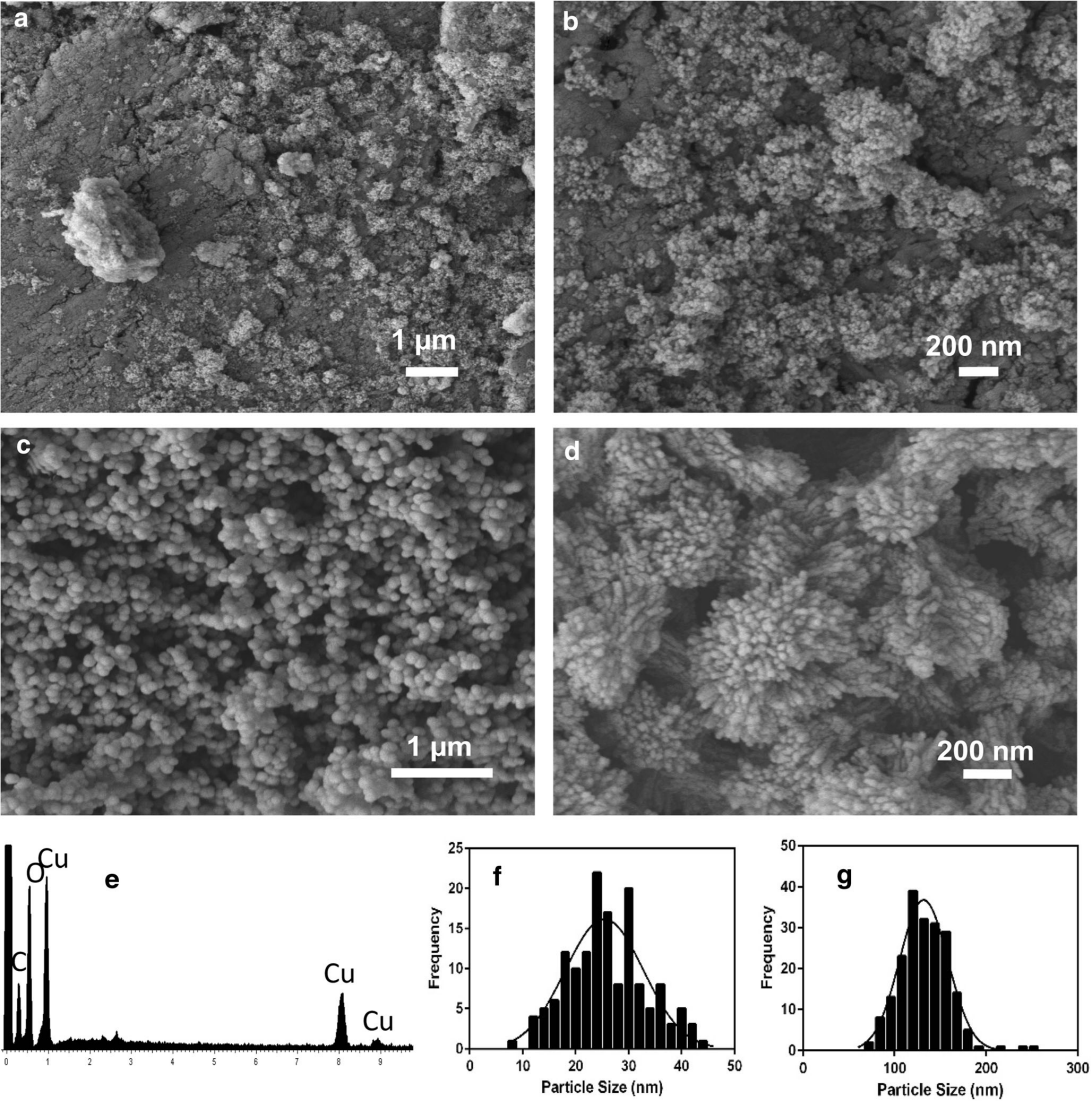
SEM images of copper carbonate minerals synthesized by **a**, **b** mixing 12-day-old *Neurospora crassa* growth supernatant with 20 mM CuCl_2_ after incubation for 16 h, and **c**, **d** mixing of 20 mM (NH_4_)_2_CO_3_ and 20 mM CuCl_2_. **e** EDXA data of the main elements in the sample synthesized from fungal growth supernatant. **f**, **g** Size distribution histograms of particles produced from fungal growth supernatant and mixing of 20 mM (NH_4_)_2_CO_3_ and 20 mM CuCl_2_ after 1 h of reaction. Typical images and spectrum are shown from several determinations

Cu-biominerals were precipitated after mixing 12-day-old *Neurospora crassa* growth supernatant with 20 mM CuCl_2_ solution. SEM results showed that the biominerals had a powder form, and the particles precipitated in this system were in the nanoscale with a mean diameter of ~ 25 nm (Fig. 1a, b). For inorganically synthesized copper carbonate minerals from reaction of (NH_4_)_2_CO_3_ and CuCl_2_ solutions, SEM showed various shapes and sizes of the copper carbonate minerals. After 1 h reaction time, the minerals produced were dispersible spherical particles with a mean diameter of ~ 130 nm (Fig. 1c). After 12 h reaction, the mineral morphology showed highly aggregated spherical agglomerates made up of monoclinic crystals (Fig. 1d). The size of the aggregates ranged from 0.5 to 5 μm in diameter.

Energy-dispersive X-ray analysis (EDXA) results showed that the main elements in the biomineral product were carbon, oxygen and copper. However, the XRPD pattern of the biominerals did not show any characteristic sharp peaks but only a broad peak which indicated the biominerals were amorphous without long-range atomic order (Fig. S1). Unlike the biominerals produced by reaction with the fungal growth supernatants, the chemical component of the inorganically synthesized copper mineral was determined as malachite (Cu_2_(OH)_2_CO_3_), as high-intensity peaks occurred at 15°, 18°, 24°, 31° and 36° corresponding respectively to the (020), (120), (220), (− 201) and (240) faces of the malachite crystals (Süsse 1967).

### Determination of fungal extracellular amino acid secretion

**Fig. 2:**
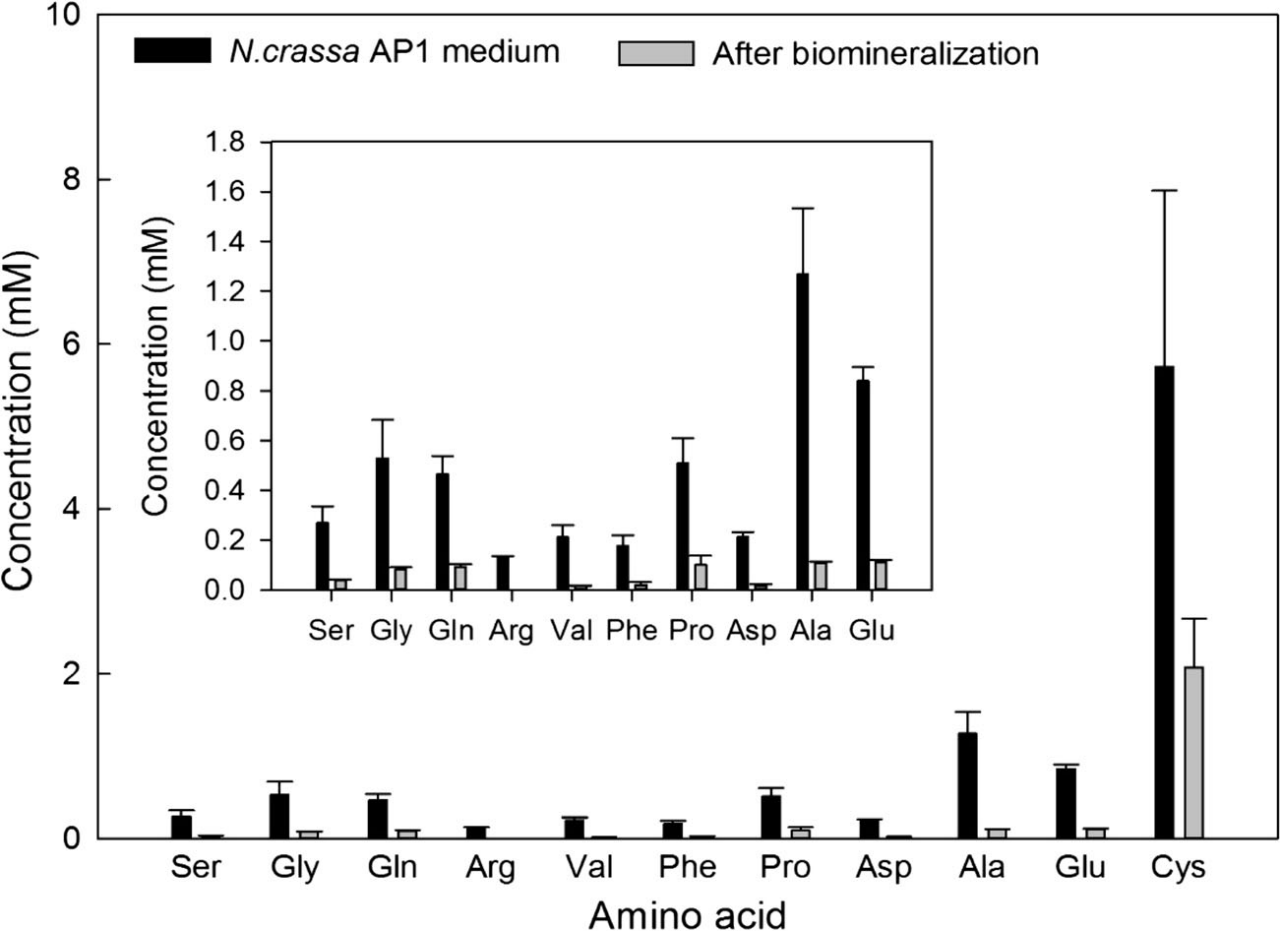
Concentrations of eleven amino acids detected in *N. crassa* culture supernatant after 12-days incubation and in the supernatant after biomineralization and removal of resultant copper carbonate precipitated. The image inset shows the concentrations of amino acids without cysteine. Analyses were conducted at least in triplicate, and the bars shown are one standard error of the mean

HPLC was used to determine the amino acids in the growth supernatant of *N. crassa* in phosphate-free AP1 medium. The results showed that 11 common amino acids were detected, including cysteine, alanine, glutamic acid, glycine, proline, glutamine, aspartic acid, valine, phenylalanine and arginine. In order to identify the amino acids potentially involved in the biomineralization of copper carbonate nanoparticles, the concentration of amino acids remaining in the supernatant after biomineralization was also measured by HPLC. The concentration of all the amino acids decreased after mineral formation which suggested the removal of amino acids from solution through association with the copper carbonate minerals (Fig. 2). For some of the amino acids detected, for example, valine, alanine and aspartic acid, more than 80% of the original amounts were removed after bioprecipitation which indicated a high affinity to bind to the biominerals (Table S1).

### Effect of amino acids on the formation of copper carbonate minerals

#### Mineral morphology

**Fig. 3:**
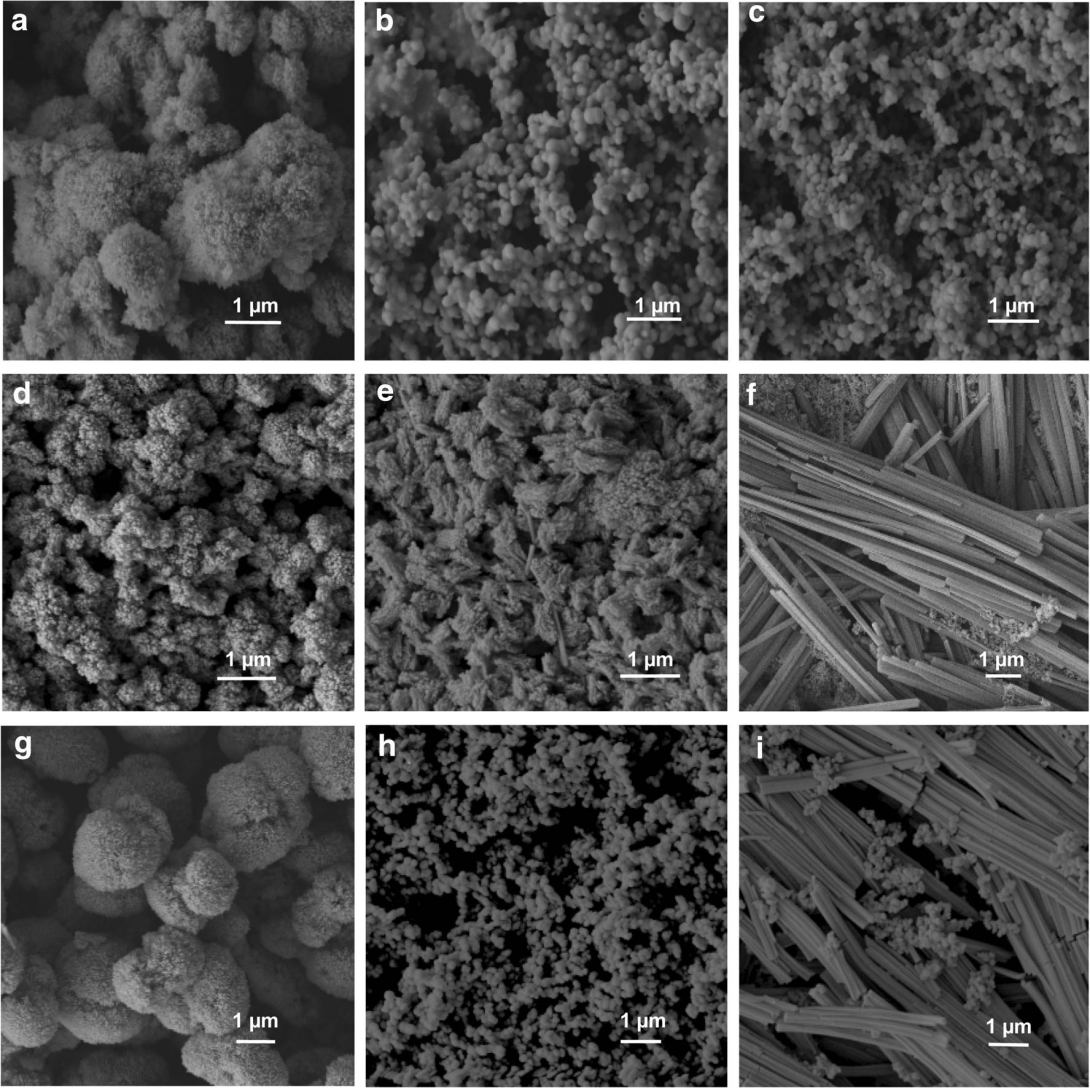
SEM images of copper carbonate nanoparticles formed with/without the addition of various amino acids. **a**–**c** 0.2, 1 and 10 mM l-glutamic acid. **d**–**f** 0.2, 1 and 10 mM l-aspartic acid. **g**–**i** 0.2, 1 and 10 mM l-cysteine, respectively. The scale bars are 1 μm, and typical images are shown from several separate determinations

The impact of the 11 amino acids that were produced by *N. crassa* on the particle morphology of the copper carbonate was investigated in preliminary studies. Among the amino acids, glutamic acid was found to prevent particle aggregation and produce nanoparticles at a 1mM concentration. Samples with different concentrations (0.2, 1 and 10 mM) of phenylalanine, arginine, valine and proline did not show much effect on nanoparticle morphology or structure. When the amino acid concentration was increased to 10 mM, some nanoparticles were produced in the presence of glutamine, alanine, serine, glycine and glutamic acid of size around 100–200 nm in diameter. We selected l-glutamic acid, l-aspartic acid and l-cysteine as target amino acids for more detailed study due to the formation of nanoparticles and unique ‘fibrous’ minerals in the presence of these amino acids (Fig. S2, Fig. S3). SEM revealed that when copper carbonate was synthesized in the presence of 0.2 mM amino acids, all the samples were spherical and formed irregularly shaped aggregates with particle size distribution ranging from ~ 0.3 to 3 μm in diameter (Fig. 3a, d, g). Aggregates growing in clusters were also observed. Each individual aggregate comprised an assemblage of many superfine particles with sizes under 100 nm. These nanoscale particles were uniform, and were tightly compacted for most of the samples, although some samples exhibited a degree of porosity, e.g. the minerals produced with 0.2 mM cysteine (Fig. 3g). With increasing concentration of amino acids, various shapes and sizes of minerals were produced. The images revealed that the particles produced in the presence of 1 mM glutamic acid were polydispersed and ranged from ~ 100 to 200 nm in diameter (Fig. 3b, Fig. S4). Unlike the spherical particle aggregates, some fibrous rod-like structures were observed in the samples with increasing concentrations of cysteine and aspartic acid (Fig. 3f, i). It was also observed that the groups of fibres showed a similar orientation, with the length usually ranging from ~ 2 to 3 μm.

#### XRPD analysis

**Fig. 4:**
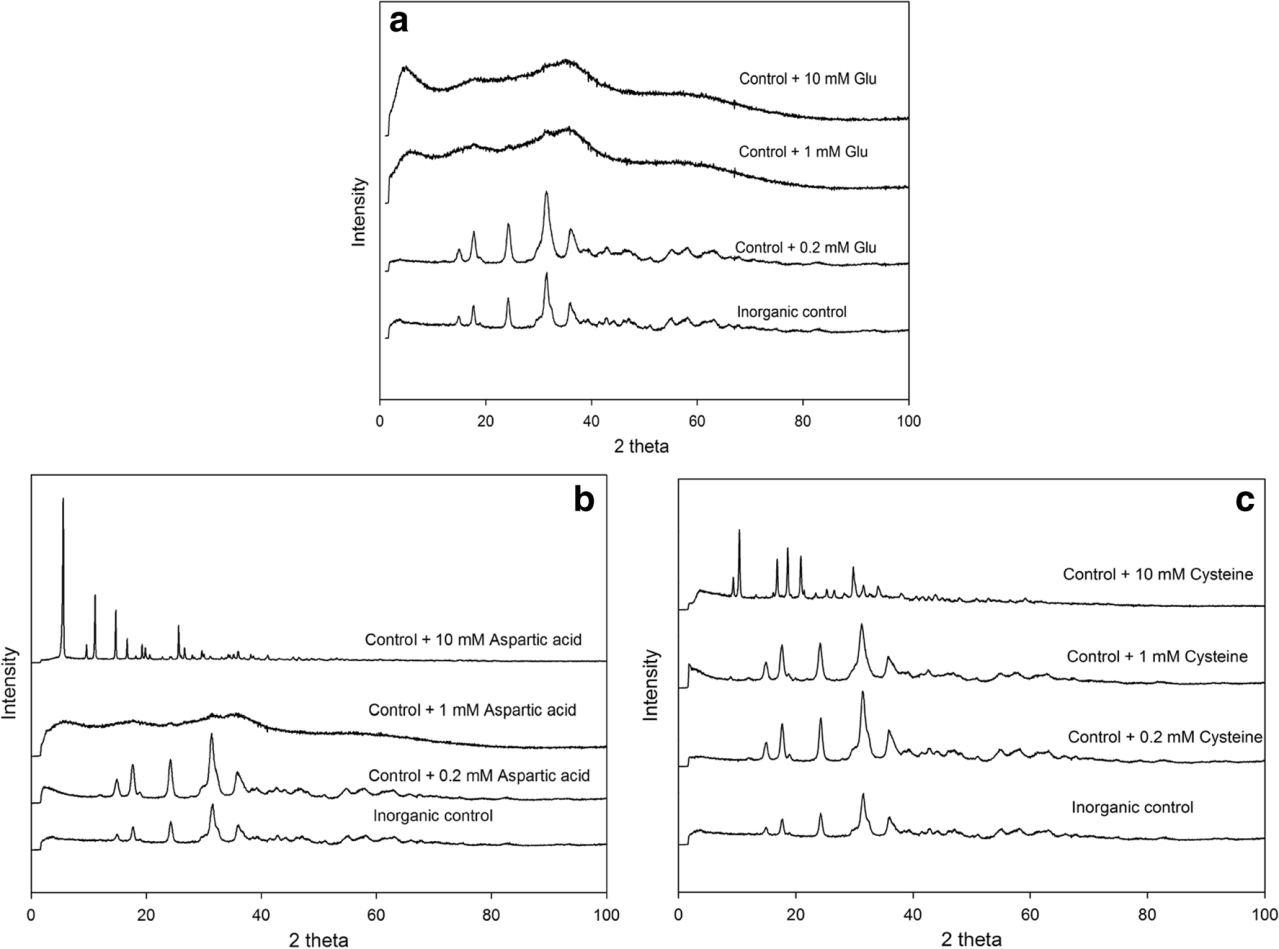
XRPD patterns of copper nanoparticles formed with/without the addition of various amino acids (**a**, l-glutamic acid; **b**, l-aspartic acid; **c**, l-cysteine) to concentrations of 0.2, 1 and 10 mM, respectively. Typical patterns are shown from several separate determinations

The XRPD pattern of copper carbonate particles synthesized inorganically confirmed them to be malachite (Cu_2_(OH)_2_CO_3_). When the concentration of added amino acid was low (0.2 mM), the chemical composition of the minerals produced in the presence of the amino acid was also determined as malachite (Fig. 4). There were some broad peaks in the XRPD patterns of the samples with 1 mM and 10 mM glutamic acid, which indicated that minerals with a very small size had been produced, based on the Scherrer equation (Patterson 1939). For the samples with 10 mM cysteine and 10 mM aspartic acid, characteristic peaks for malachite, and other inorganic copper-bearing minerals were not detected. Some very finely crystallized minerals were formed, and minerals with a unique fibrous structure were observed for these two samples. Some promising results were obtained from the XRPD database, as patterns for C_20_H_18_CuN_4_O_6_ (00-705-2616) and C_33_H_30_Cu_3_O_21_2CH_3_OH (00-151-4153) were very similar to the XRPD patterns of the samples with cysteine and aspartic acid, therefore suggesting the formation of complexes between copper and the amino acids.

#### FTIR spectral analysis

**Fig. 5:**
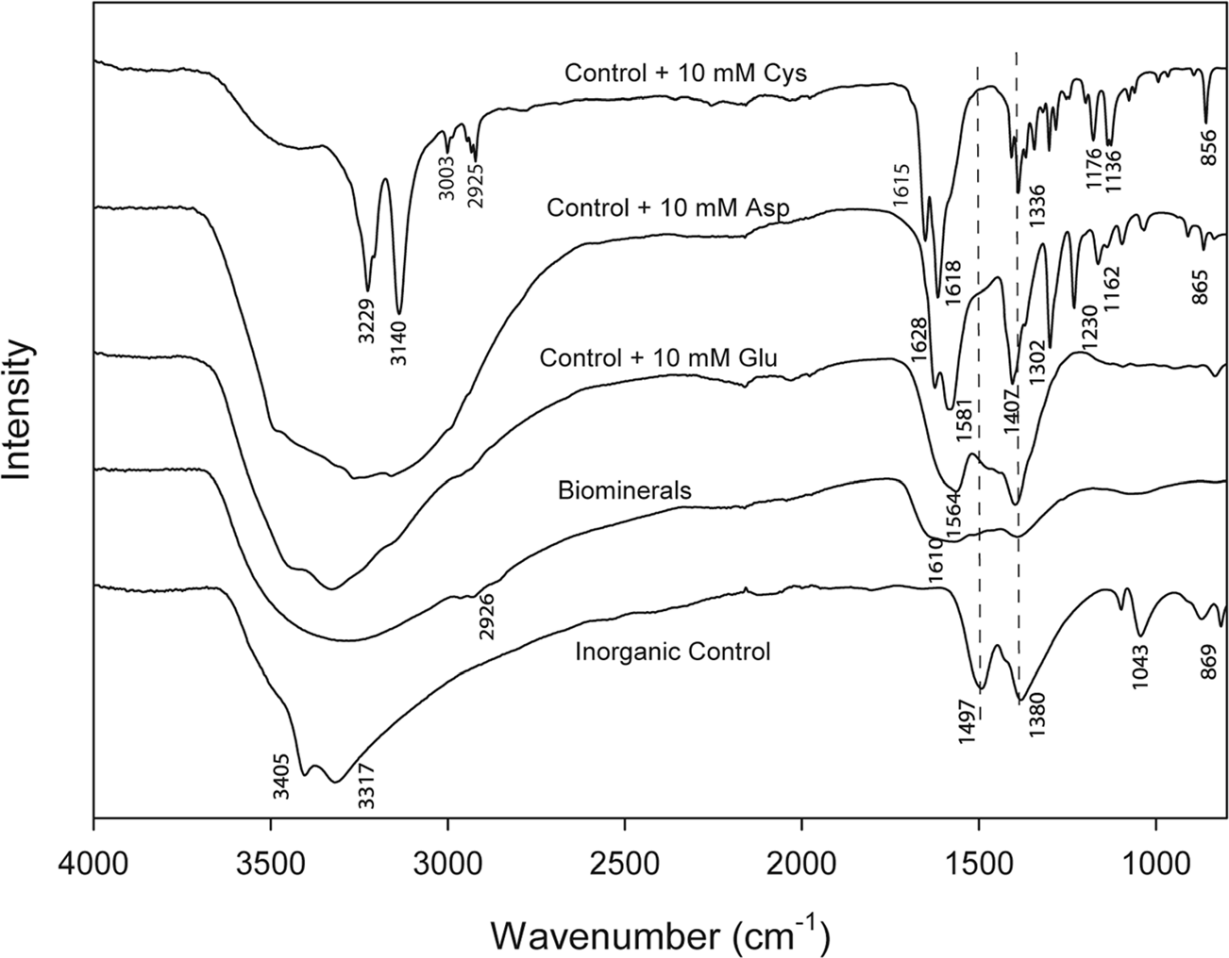
FTIR transmittance spectra of synthesized inorganic malachite (control), copper carbonate minerals precipitated from *N. crassa* growth supernatant (biominerals) and copper minerals produced in the presence of 10 mM l-glutamic acid, l-aspartic acid and l-cysteine. A typical spectrum is shown

ART-FTIR was conducted to identify possible interactions between the copper minerals and organic additives, as amino acids could be incorporated into the mineral structure during crystal growth. FTIR spectra can also provide additional identification for the amorphous minerals which showed no discernible patterns in XRPD analysis. A broad band was observed at around 3400 cm^−1^ which represented the stretching vibration of intermolecular bonded OH groups (Fig. 5). Based on previous studies, some characteristic peaks of the CO_3_^2−^ group at 1497 and 1380 (asymmetric stretch), 1043 (symmetric stretch) and 869 cm^−1^ (bending mode) were present in the spectrum of the inorganic control which again confirmed the formation of copper carbonate (Liu et al. 2016). For the samples produced from the fungal growth supernatant and those produced in the presence of glutamic acid, which showed amorphous patterns in XRPD analysis, FTIR patterns became broader and less sharp as atomic disorder increased. FTIR patterns of biominerals and mineral produced in the presence of 10 mM glutamic acid were very similar with malachite, with characteristic peaks of CO_3_^2−^ group at 1497 and 1380 cm^−1^, suggesting the formation of carbonate minerals. The peak at 1497 cm^−1^ shifted towards a higher wavelength in all biomineral and amino acid samples. At this region, the bending of N-H (~ 1600 cm^−1^) of amines and the stretching vibration of the CO_3_^2−^ group (1497 cm^−1^) overlapped. This might be attributable to hydrogen bonding between the carboxyl groups of amino acids and the oxygen of the carbonyl groups, indicating the molecular interaction between amino acids and carbonate minerals (Ghadiri et al. 2014). In addition, for the biomineral sample, peaks in the 2920 to 2853 cm^−1^ region represent the asymmetric and symmetric stretching vibration of the C-H of alkanes, respectively, which is evidence for the association of organic materials secreted by the fungus into the mineral structure (Xu et al. 2012). For the samples with aspartic acid and cysteine, very strong N-H bending peaks (~ 1622 cm^−1^) can be determined, as well as some other organic peaks belonging to amino acids (Table 1). The characteristic carbonate peaks were absent in the spectrum of the sample produced in 10 mM cysteine. Significantly, cysteine usually shows a strong peak near 2540 cm^−1^, which confirms the presence of thiol groups (-SH) in the cysteine molecule (Pawlukojć et al. 2005). This peak disappeared in the spectrum of the sample produced in the presence of 10 mM cysteine, suggesting the thiol group of cysteine was associated within minerals through the formation of Cu-S bonds. These results indicate the formation of Cu-aspartate and Cu-cysteine complexes.

**Table 1:** FTIR data of copper complexes produced in the presence of 10 mM aspartic acid (Cu-asp) and 10 mM cysteine (Cu-cys), respectively

Cu-asp complexes Wavelength (cm^−1^)	Assignments (Navarrete et al. 1994)	Cu-cys complexes Wavelength (cm^−1^)	Assignments (Pawlukojć et al. 2005)
865	CC_str._	856	CC_str._
910	CH_2rock._	1076	NH_3rock._
1097	CN_str._	1136	CH_bend._
1162	NH_3rock._	1176	CH_2twist._
1230	CH_2twist._	1302	CH_2wagg._
1302	NH_3rock,_	1388	CO_2sym._
1407	CO_str._	1579	NH_3bend._
1584	CO_2str._	1618	NH_3bend._
1628	NH_3bend._	1651	CO_2str._
		2925	CH_str._
		3003	CH_2str._
		3140	NH_3str._
		3229	NH_3str._

#### X-ray photoelectron spectroscopy

**Fig. 6:**
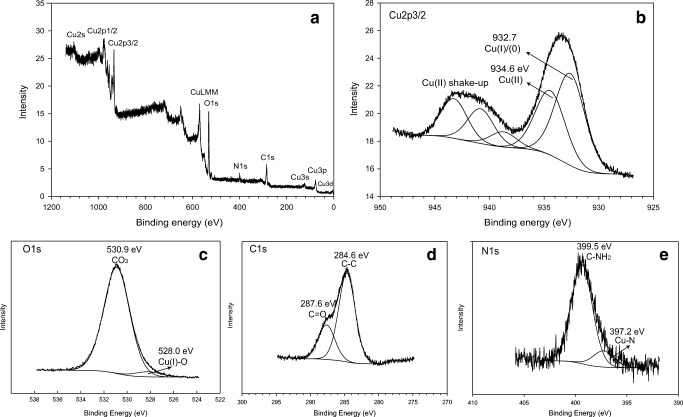
XPS spectra of copper carbonate minerals precipitated from *N. crassa* growth supernatant. **a** Total spectra and **b**–**e** XPS Cu2p, O1s, C1s and N1s spectra. A Typical spectra are shown

X-ray photoelectron spectroscopy (XPS) was performed to study the surface chemical properties of Cu carbonate precipitates derived from the fungal growth supernatant. XPS spectra over a binding energy range of 1200–0 eV (Fig. 6a) showed the presence of C, N, O and Cu on the biomineral samples. The presence of N was attributed to organic components that bound to the mineral surface, for example, secreted amino acids and proteins. The valence states of the biominerals were further characterized by high-resolution XPS spectra as shown in Fig. 6b–e. Some ‘shake-up’ satellite peaks at binding energies of 940~945 eV shown in the high-resolution Cu2p3/2 spectrum are characteristic features of Cu(II), which indicates the presence of various Cu(II) species based on the complexity of the shake-up structures (Fig. 6b). Cu 2p main peaks could be fitted into two components at 934.6 eV and 932.7 eV, which are assigned to Cu(II) and Cu(I)/Cu(0), separately (Biesinger 2017). Both metal carbonates and organic C-O bonds give a O1s contribution at the approximate same binding energy (Stoch and Gablankowska-Kukucz 1991), and the complexity of various Cu(II)-containing minerals (e.g. Cu_2_CO_3_(OH)_2_, Cu(OH)_2_, Cu complexes) makes assignment of the O1s region more complex (Fig. 6c). These factors could account for the downward shift of the O1s peak (~ 531 eV) compared with the O1s peak detected for standard malachite (531.5 eV) (Wagner et al. 1980). The C1s spectrum could be deconvoluted into two individual peaks with binding energies of 284.6 and 287.6 eV (Fig. 6d). The peak at 284.6 can be assigned to non-oxygenated C-C or C-H bonding (Choi et al. 2009). The peak at 287.6 eV could be the carbonate peak shifting to a lower binding energy, which can be attributed to the interaction of carbonate with lower positive oxidation state bonding, for example, amine C-N bonding (286 eV) (Tawil et al. 2013) or possibly the formation of bicarbonate HCO_3_^−^. The N1s peak could not be fitted with a single symmetrical component. Besides the C-NH_2_ bond at 399.5 eV, a low-intensity component at a binding energy of 397.2 eV was also found in the spectrum (Fig. 6e), which is believed to be Cu-N bonding (Meng et al. 2017). The formation of Cu-N could be an indicator of the reaction of organics with Cu, leading to the association of N on the surface of the biominerals.

#### Speciation modelling using Geochemist’s Workbench

**Fig. 7:**
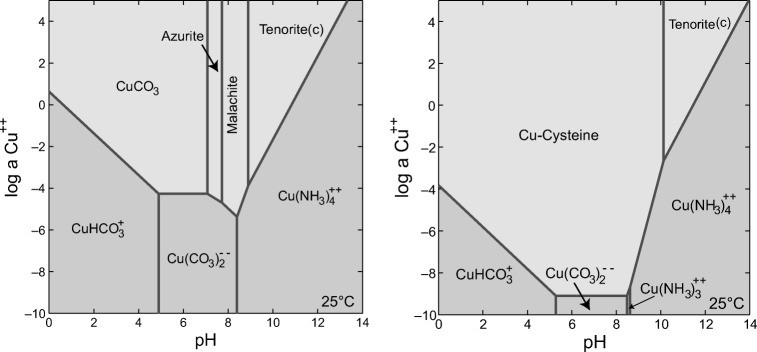
Solubility diagrams of Cu^2+^ versus pH at 25 °C for (left) biomineralization in a simulated AP1 fungal growth supernatant and (right) the reaction of inorganic (NH_4_)_2_CO_3_ and CuCl_2_ in the presence of 20 mM cysteine

The stability of copper minerals was assessed using Geochemist’s Workbench (GWB) by modelling the predominance of chemical species in the fungal growth supernatant in order to determine the chemical components of the biomineralization products. The pH of *N. crassa* growth supernatant was around pH 8.5 after 12-day incubation. Based on the solubility diagram, when the concentration of Cu^2+^ was 20 mM, the only possible mineral precipitated in this system was malachite (Fig. 7). The modelling results for the reaction of copper and cysteine showed the formation of Cu-cysteine complexes at pH = 8.5. Compared with the *N. crassa* growth supernatant, it was therefore not possible for malachite (Cu_2_(OH)_2_CO_3_) to be precipitated in the presence of cysteine.

#### Thermal stability analysis

**Fig. 8:**
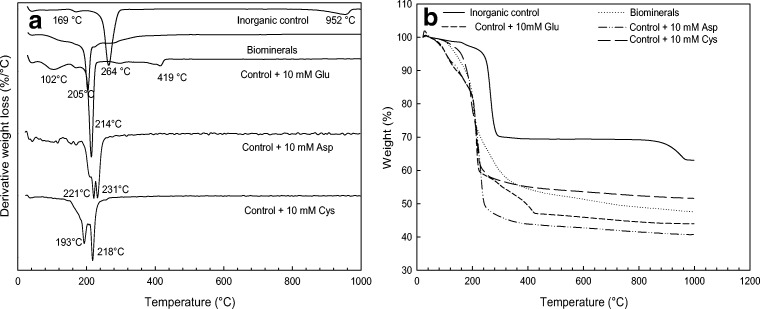
**a** Differential thermal analysis (DTA) of synthesized inorganic malachite (control), copper carbonate minerals precipitated from *N. crassa* growth supernatant (biominerals) and copper minerals produced in the presence of 10 mM l-glutamic acid, l-aspartic acid and l-cysteine; **b** shows the thermogravimetric analysis (TGA) of the same samples

The association of amino acids, and other organic materials produced by the fungus, with the minerals was also confirmed by thermogravimetric analysis (TGA). Representative derivative thermogravimetric analysis (DTA) and TGA profiles are shown in Fig. 8. The samples containing organic matter, including the minerals produced by biomineralization and those precipitated in the presence of amino acids, showed more weight loss compared with the inorganically synthesized minerals. Organic material is decomposed at 600 °C, and the percentage weight loss between 75 and 600 °C of the control sample, biomineral and samples obtained in the presence of 10 mM Glu, Asp and Cys was 30.2%, 48.3%, 52.8%, 55.6% and 45.9%, respectively. This calculation allows for an initial mass loss due to the loss of physically adsorbed water from 60 to 75 °C. The decomposition of organic materials which were associated with the minerals, i.e. amino acids, contributed to the additional weight loss compared with the control sample. Specifically, it can be seen that the sample with 10 mM aspartic acid showed the highest weight loss, which was 22.3% greater than the inorganic control samples. In spite of losing physically adsorbed water, control abiotically synthesized malachite showed three decomposition peaks, at 169, 264 and 952 °C, which can be attributed to the loss of OH, decomposition of copper carbonate and the loss of the oxygen of copper oxide in an inert atmosphere, respectively (Ding et al. 2002; Frost et al. 2002). The peaks around 100–200 °C were related to the decomposition of amino acids. For the samples with glutamic acid/biominerals, the peaks present at the temperature around 250–300 °C can represent decomposition of the amino acids which were intercalated between the molecular structures of the minerals. Amino acids can be decomposed easily at temperatures around 200 °C (Rodante and Marrosu 1990). Intercalated amino acids inside mineral structures can be protected against decomposition therefore showing a relatively higher decomposition temperature. The peaks of copper carbonate decomposition (around 264 °C) shifted towards a lower temperature in the glutamic acid/biomineral samples which indicated that the decomposition of amino acids leads to an obvious decrease in the thermal stability of the malachite. For the sample with 10 mM glutamic acid, the temperature for loss of oxygen decreased from 947 to 419 °C. For the samples produced in the presence of aspartic acid and cysteine, two strong signals were observed around 190 to 230 °C, suggesting the decomposition of Cu-amino acid complexes.

XRPD analysis of the final products after thermal decomposition (Fig. S5) showed that different copper-bearing minerals resulted. For the inorganic control, cuprite (Cu_2_O) was produced. The biominerals, the sample with 10 mM glutamic acid and 10 mM aspartic acid showed the production of metallic copper after TG analysis. It is therefore suggested that the copper carbonate minerals containing organic material were more easily reduced to metallic copper compared to the inorganic controls, and they may also possess less thermal stability. TGA of the samples produced with 10 mM cysteine gave pure phase chalcocite (Cu_2_S).

## Discussion

The copper carbonate nanoparticles formed by a fungal-mediated biomineralization reaction showed an amorphous powder form without any clear crystallinity, and this result was also consistent with XRPD data as no distinguishable peaks in XRPD patterns were observed for the amorphous minerals in the absence of any ordered atomic structure. Although the GWB modelling and FTIR results suggested that basic copper carbonate, malachite (Cu_2_(OH)_2_CO_3_), was formed under the experimental conditions, the presence of amino acids and other organic molecules secreted from fungi can affect the purity and valence state of the Cu mineral. The Cu2p3/2 XPS bands appeared at around 932 eV which was assigned to either Cu(I) or Cu(0) species, indicating the reductive Cu species formed via some complex-forming reactions inside the structure of the copper carbonate minerals. Our XPS results further suggested the Cu complexes were formed by bonding the N atoms of organic molecules with the Cu species (Cu-N) on the mineral surface. Liu et al. (2016) studied the reaction of a novel organic surfactant HATT (which has amino and thione functional groups) with malachite, and showed that HATT was associated with malachite by the formation of Cu-N and Cu-S surface complexes. It is not unusual that Cu(II) can be reduced to Cu(I) and form Cu(I) and/or Cu(II) complexes with amino acids, e.g. glycine (Cedzynska et al. 1981) and cysteine (Rigo et al. 2004). Redox reactions of Cu are also involved in many enzymatic processes, for example the functions of copper chaperone and copper transport proteins (Valentine and Gralla 2002). Some previous studies showed that cysteine and tyrosine residues played a key role in the reduction of Cu(II) to Cu(I) by some copper-binding proteins (Opazo et al. 2003).

Nanoparticles produced from the biomineralization reaction showed the smallest particle size (mean diameter around 26 nm), compared to the inorganically synthesized sample and samples obtained in the presence of various amino acids. Nucleation and growth of nanoparticles is closely associated with the microenvironment of the specific particle system (Thanh et al. 2014). The reaction conditions, concentration of solute and particle surface charge, as well as the presence of organic matter can have an important influence on the final morphology of such particles. Since the fungal growth supernatant contained various metabolites, including free amino acids, proteins and extracellular polymeric substances (EPS), which can act as stabilizing agents in nanoparticle formation (Tourney and Ngwenya 2014), such biomolecules may be bound and subsequently block growth sites on the surface of copper carbonate particles after ‘burst-nucleation’. The mean particle size (*D*) is related to both the particle growth rate and the nucleation rate:

5 $$ D=1.203{\left(G/N\right)}^{1/3} $$

where *G* refers to the particle growth rate and *N* is the nucleation rate (Byrne et al. 2011). When the growth rate of nuclei is inhibited, the resultant particle size is smaller, and this explains the deficient development of crystal structure and the formation of amorphous, nanoscale particles.

The copper carbonate precipitated in the presence of various concentrations of different amino acids showed different morphologies compared with abiotically synthesized copper carbonate particles produced from mixture of 20 mM (NH_4_)_2_CO_3_ and 20 mM CuCl_2_. The mean size of the inorganic control copper carbonate particles in the absence of any additives was ~ 130 nm in diameter after 1 h reaction time, and these crystals aggregated together to form microscale particles after 12 h. Nanoparticles have a high surface area to volume ratio, and the total free energy of nanoparticles tends to decrease on reducing the interfacial area to maintain the stability of the system. This usually results in particle agglomeration or recrystallization. One of the most remarkable features of these minerals was the uniformity of the nanocrystals within the aggregates which all had the same morphology and size, and this suggested that the nanoparticles nucleated simultaneously and grew at the same rate before aggregation (Kwon and Hyeon 2008). When various amino acids were added into the chemical reaction of copper carbonate, the incorporation of amino acids within the mineral structure was confirmed by FTIR analysis. Among all the amino acids tested, the reaction with 10 mM glutamic acid produced the smallest sized particles, and these particles were stabilized in the early phase of crystal growth and prevented from aggregation with only 1 mM glutamic acid. It is reported that the point of zero charge (pzc) of malachite is around pH 7.6, and malachite also has a high positive charge, ~ 20 mV on the surface at a neutral pH (Saha and Das 2009). Therefore, it can be concluded that the negatively charged amine group side chain of glutamic acid showed a high affinity for binding to positive mineral surfaces (Wang et al. 2015; Wolthers et al. 2008; Zare et al. 2013). Whether particles stay dispersed or aggregated in a system is controlled by repulsive and attractive forces between particles, for example, gravity, electrical charge and van der Waals forces (Adamczyk and Weroński 1999). It is therefore suggested that the Glu-associated particles with a negatively charged surface would have a stronger repulsive force than an attractive force which would make the particles more dispersible and also inhibit their further growth. The mean size of particles synthesized in the presence of 10 mM Glu was ~ 130 nm in diameter, which was smaller than those produced in the presence of 1 mM Glu (mean diameter ~ 160 nm). It is suggested an increasing concentration of the added amino acid can decrease the activation energy for nucleation, increase the rate of nucleation and decrease the size of the crystals (Mann 2001).

For acidic amino acids like aspartic acid, it was found that these amino acids resulted in the formation of rod-like crystals. Long fibrous crystals were also observed in the samples with 10 mM cysteine. Peptides and amino acids can interact with divalent transition metals, and form ‘complexes’ that have received significant attention, especially the copper complexes of amino acids (Kryukova et al. 2005). The synthesis of such complexes via complexation and chelation also highlights the possibility of artificial mimics of metal-containing enzymes for use as catalysts. Previous work reported using the copper-cysteine complexes to enhance photocatalytic H_2_ production by 150 times compared with CdSe catalyst (Peng et al. 2015). In our reaction system, as shown by the GWB modelling results, the amino and carboxylate groups of aspartic acid and cysteine can chelate copper ions preferentially compared with carbonate ions, which will further prevent the formation of copper carbonate minerals (Sóvágó et al. 2012). The oriented nucleation and growth of minerals, which enhanced growth in specific directions but limited growth rates in other directions, resulted in formation of minerals with rod-like or fibrous structure. The formation of Cu_2_S from thermolysis of Cu-cysteine complexes confirmed the breakup of thiol group and formation of Cu-S bonding, which can shed some light on the production of copper sulphide with impressive electrocatalytic properties (Choi et al. 2009).

Biominerals that exist in natural and synthetic environments show a variety of morphologies and structures, but the effect of small biomolecules, such as amino acids, on the biomineralization process remains poorly understood. This work demonstrates the extracellular production of well-dispersed copper nanoparticles by using biomass-free urea-grown *Neurospora crassa* growth supernatants. The nanoparticles exhibited a spherical morphology with a mean diameter around 25 nm, which was much lower than inorganically synthesized particles or particles produced solely in the presence of amino acids. Eleven different amino acids were secreted by *Neurospora crassa*, and among these, glutamic acid was found to stabilize the particles in the early phase of growth and prevented them from aggregating, even at a low concentration (1 mM). FTIR revealed the molecular interaction of amino acids and copper minerals, confirming the association of organic substances secreted by the fungus into the mineral structure. Thermal treatment of copper carbonate biominerals also suggested a facile method for producing Cu, Cu_2_O and Cu_2_S mineral products. Overall, this work provides further understanding of the potential application of fungal system for nanoparticle synthesis, the significance of amino acids in microbially induced carbonate biomineralization and possible means of controlling particle size and aggregation, and the further production of useful metal and biomineral products.

## Electronic supplementary material

ESM 1 (PDF 2.59 mb)
