# Supplementary material for: Amino acid secretion influences the size and composition of copper carbonate nanoparticles synthesized by ureolytic fungi
Source: Appl Microbiol Biotechnol. 2019 Jul 9;103(17):7217–30. doi: 10.1007/s00253-019-09961-2 (PMC6691030; doi:10.1007/s00253-019-09961-2)
Supplement: Supplementary file 1 — (PDF 2.59 mb) [file 253_2019_9961_MOESM1_ESM.pdf]

Applied Microbiology and Biotechnology

Amino acid secretion influences the size and composition of copper carbonate nanoparticles synthesized by ureolytic fungi

Feixue Liu [1], Laszlo Csetenyi [2], Geoffrey Michael Gadd [1,*]

[1] *Geomicrobiology Group, School of Life Sciences, University of Dundee, Dundee, DD1 5EH, Scotland, United Kingdom*

[2] *Concrete Technology Group, Department of Civil Engineering, University of Dundee, Dundee, DD1 4HN, Scotland, United Kingdom*

[*] For correspondence. E-mail: g.m.gadd@dundee.ac.uk; Tel. +44 1382384767.

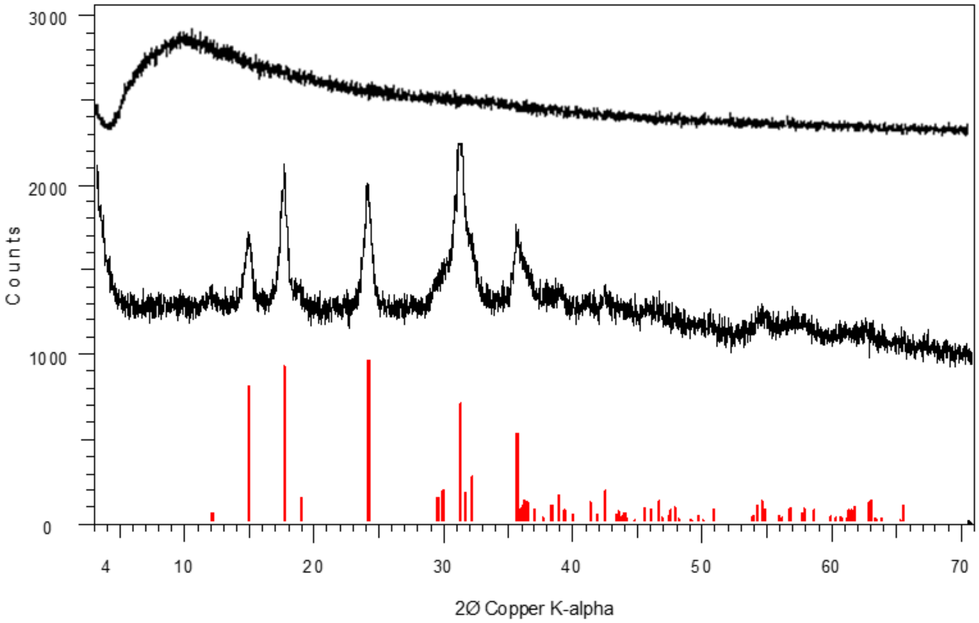

Fig.S1 X-ray powder diffraction patterns of copper carbonate nanoparticles synthesized by mixing 12d-old *Neurospora crassa* growth supernatant with 20 mM $CuCl_2$ after incubation for 16 h (top) and well-crystallized inorganically synthesized malachite (middle) obtained from mixture of 20 mM $(NH_4)_2CO_3$ and 20 mM $CuCl_2$. The bottom pattern is the diffraction pattern of standard malachite. Typical patterns are shown from several determinations.

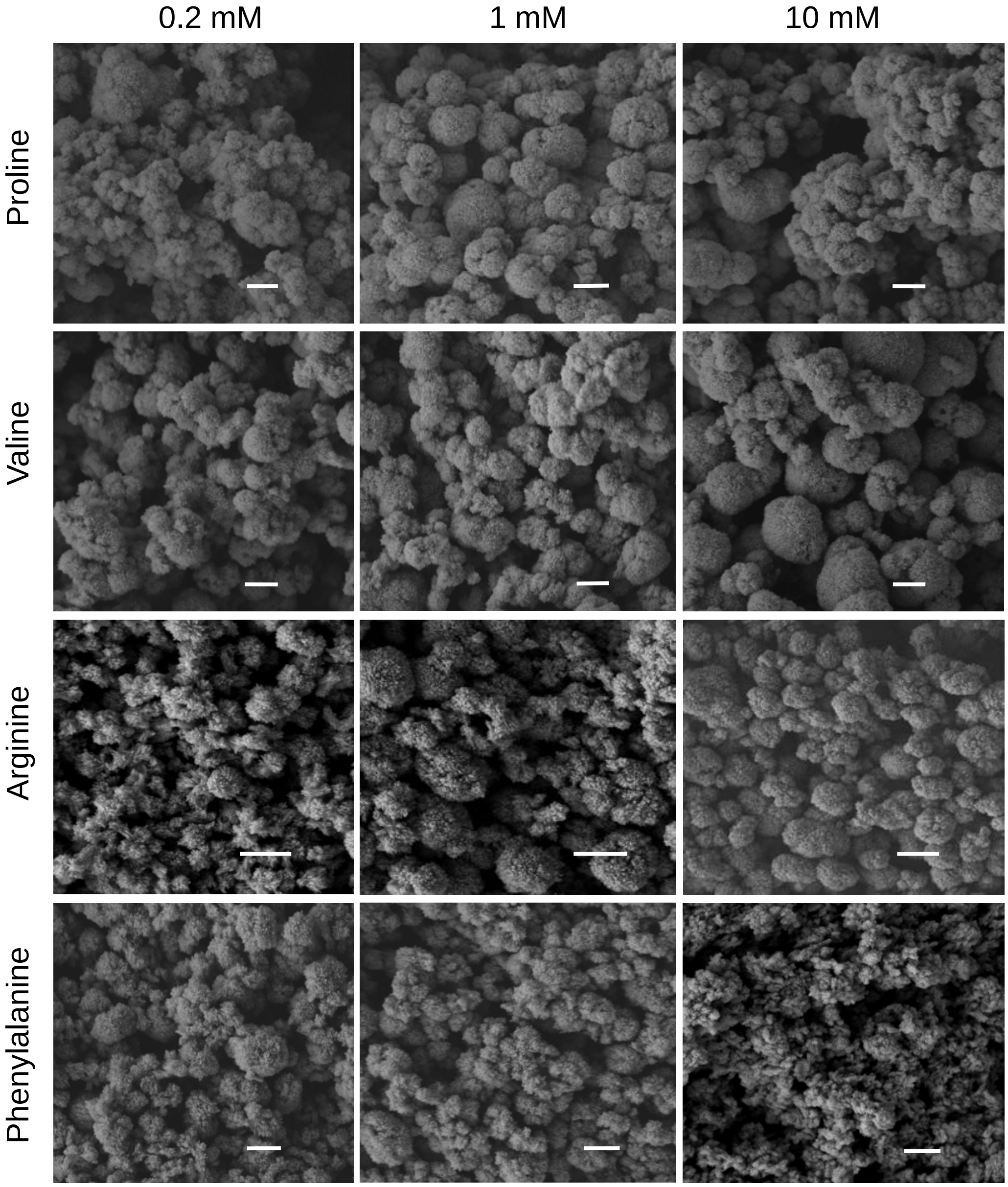

Fig. S2 SEM of copper-bearing minerals precipitated by mixing of 20 mM $(NH_4)_2CO_3$ and 20 mM $CuCl_2$ in the presence of 0.2 mM, 1 mM and 10 mM phenylalanine, arginine, valine and proline. The scale bars are 1 μm. No obvious change of mineral morphology was found in the presence of different concentrations of amino acids.

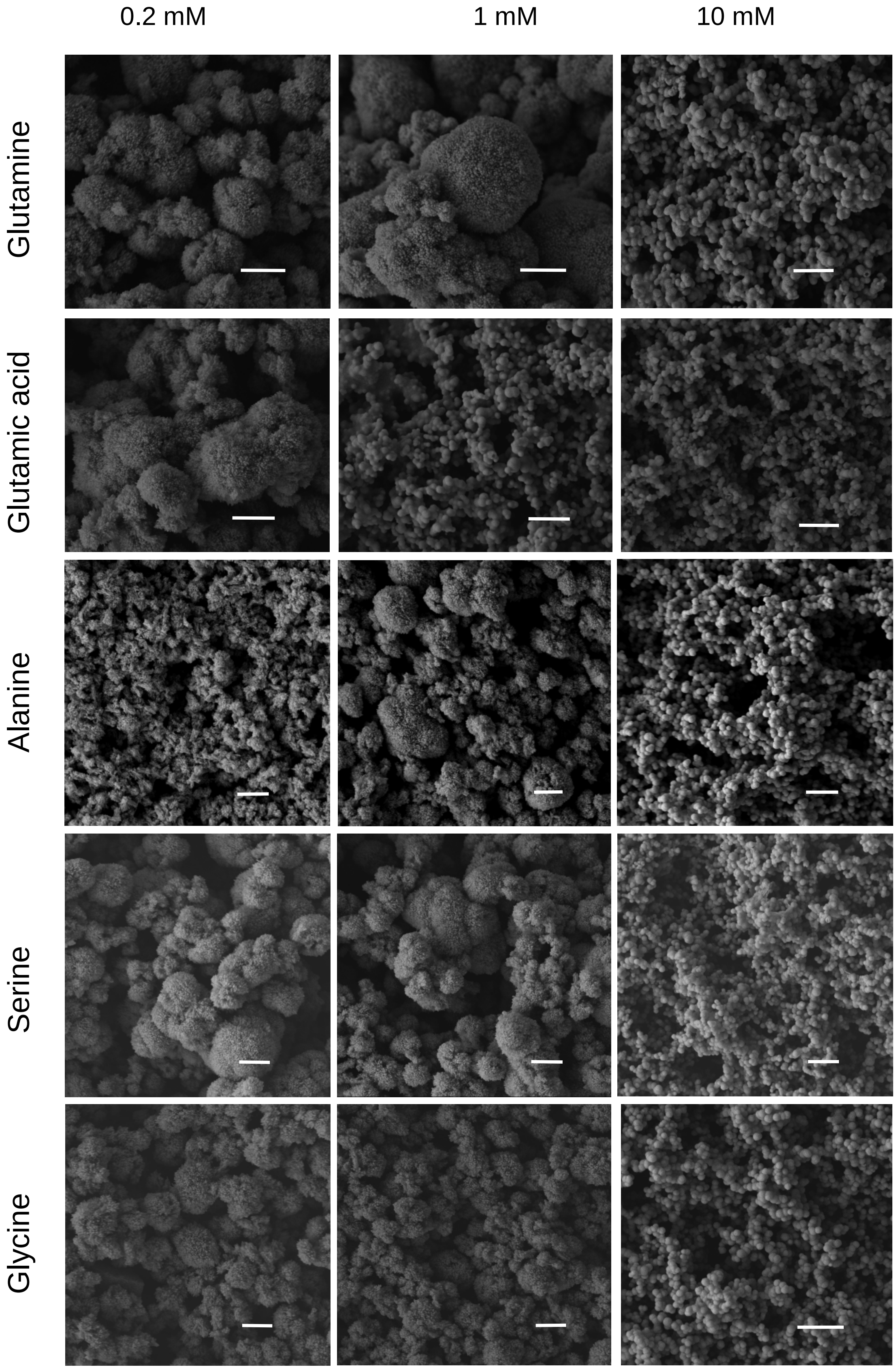

0.2 mM
1 mM
10 mM
Glutamine
Glutamic acid
Alanine
Serine
Glycine

Fig. S3 SEM of copper-bearing minerals precipitated by mixing of 20 mM $(NH_4)_2CO_3$ and 20 mM $CuCl_2$ in the presence of 0.2 mM, 1 mM and 10 mM glycine, serine, alanine, glutamic acid and glutamine. The scale bars are 1 µm. Nanoparticles with size ranging from 100-200 nm in diameter were formed in the presence of 1mM glutamic acid and 10 mM glycine, serine, alanine and glutamine. Therefore glutamic acid, aspartic acid and cysteine were selected as target amino acids for more detailed study due to the formation of nanoparticles with a low concentration of amino acid (1 mM) and unique 'fibrous' minerals in the presence of those amino acids.

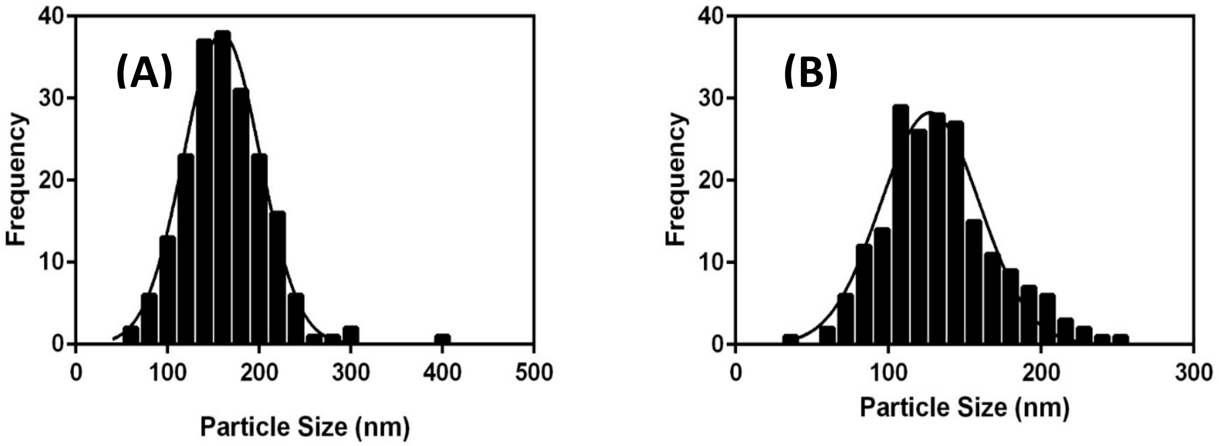

Fig. S4 Size distribution histograms of copper carbonate nanoparticles produced in the presence of 1mM glutamic acid (A) and 10 mM glutamic acid (B).

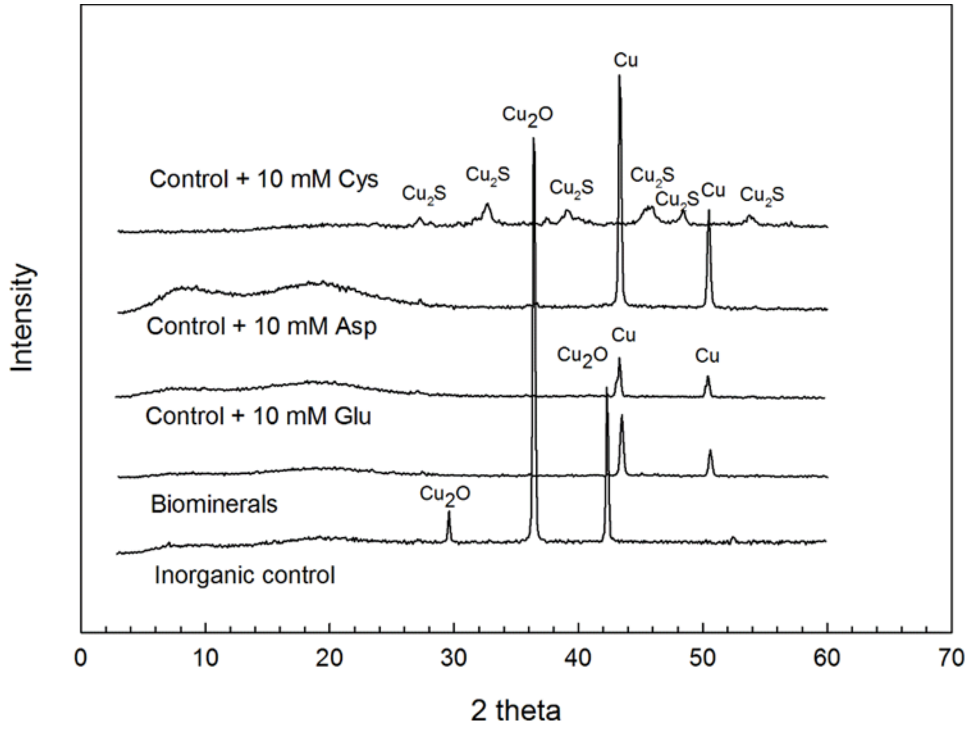

Fig. S5 XRPD analysis of the products of thermal decomposition of the copper minerals produced under various conditions: synthesized inorganic malachite (control), copper carbonate minerals precipitated from N.crassa growth supernatant (biominerals) and copper minerals produced in the presence 10 mM L-glutamic acid, 10mM L-aspartic acid and L-cysteine.

Table S1 Concentrations of eleven amino acids detected in 12-d old *N. crassa* culture supernatant, and in the supernatant after copper carbonate biomineralization, with the removal efficiency (%) for each amino acid. Values are ± one standard error and samples were conducted at least in triplicate.

Amino acid	Abbreviation	Initial conc.(mM)	After reaction (mM)	Removal efficiency (%)
Valine	Val	0.212 ± 0.047	0.015 ± 0.002	85.85
Aspartic acid	Asp	0.213 ± 0.020	0.017 ± 0.006	84.04
Alanine	Ala	1.268 ± 0.266	0.108 ± 0.005	82.97
Phenylalanine	Phe	0.177 ± 0.042	0.021 ± 0.010	76.27
Glutamic acid	Glu	0.841 ± 0.054	0.110 ± 0.010	73.84
Serine	Ser	0.268 ± 0.067	0.038 ± 0.004	71.64
Glycine	Gly	0.530 ± 0.154	0.082 ± 0.008	69.06
Proline	Pro	0.509 ± 0.102	0.100 ± 0.037	60.71
Glutamine	Gln	0.465 ± 0.074	0.092 ± 0.011	60.43
Cysteine	Cys	5.729 ± 2.136	2.080 ± 0.588	35.01
arginine	Arg	0.130 ± 0.006	n.d.	n.d.

**** n.d – not detected: the concentration was below the detection limit.***
